# Nephrotic and Non-Nephrotic Focal Segmental Glomerulosclerosis: Clinical Characteristics, Etiology, and Columbia Classification

**DOI:** 10.3390/diagnostics15020120

**Published:** 2025-01-07

**Authors:** Gabriel Figueiredo, Luis Yu, Lectícia Barbosa Jorge, Viktoria Woronik, Cristiane Bitencourt Dias

**Affiliations:** 1 Nephrology Department, Hospital das Clínicas of São Paulo, São Paulo 05403-900, Brazilluisyu@usp.br (L.Y.);; 2 Laboratory of Renal Pathophysiology, Hospital das Clínicas, Faculty of Medicine, University of São Paulo, São Paulo 05508-220, Brazil

**Keywords:** focal segmental glomerulosclerosis, nephrotic syndrome, proteinuria, kidney failure

## Abstract

**Introduction**: Focal segmental glomerulosclerosis (FSGS) is a pattern of kidney injury with diverse causes and pathogeneses, resulting in podocyte injury and depletion. It can be classified as primary, genetic, or secondary. Because FSGS classically has a worse prognosis in patients with nephrotic syndrome, most studies have focused on the treatment and evolution of these patients, resulting in a lack of data related to patients without nephrotic syndrome. The objective of this study was to establish the main etiologies, characteristics, and evolution of renal disease in FSGS patients with nephrotic and non-nephrotic proteinuria. **Methods**: This was a retrospective, single-center study that included 140 patients with a biopsy-confirmed diagnosis of FSGS in the 2009–2017 period. Patients were separated into those with and those without nephrotic syndrome at diagnosis, and these two groups were compared in terms of the clinical characteristics, histological profile, and outcome. Non-nephrotic patients with unfavorable progression were selected for ultrastructural analysis with electron microscopy. **Results**: During the study period, 32.9% of the patients with FSGS had non-nephrotic proteinuria at diagnosis. This group had a larger proportion of patients with hypertension and a not otherwise specified FSGS variant on histology. The proportion of patients with secondary forms of FSGS was comparable between the two groups, with HIV infection and systemic lupus erythematosus being predominant. Progression to renal replacement therapy occurred in 31.3% of the patients in the nephrotic group and in 26.8% of those in the non-nephrotic group, with no statistical difference between them. All of the non-nephrotic group patients who progressed to renal replacement therapy were analyzed by electron microscopy, the diagnosis of FSGS was confirmed, and there was the finding of high chronicity in these patients. **Conclusions**: Among patients with FSGS, those without nephrotic syndrome had a poor renal outcome at a frequency similar to that of those with nephrotic syndrome. Factors related to better renal survival were having had a complete response to treatment in the case of those with nephrotic syndrome and having achieved proteinuria values of less than 1.5 g/day in the case of those without nephrotic syndrome.

## 1. Introduction

Focal segmental glomerulosclerosis (FSGS), one of the main glomerulopathies that leads to chronic kidney disease [1,2], has recently been increasing in incidence, as reported in studies conducted in the United States [3,4] and Brazil [5,6]. In FSGS, the histological pattern is represented by a matrix increase with the obliteration of the capillary lumen in at least one glomerulus [7]. The sclerotic lesion initially affects portions of the glomerular tuft (segmental involvement) and part of the glomerulus (focal involvement), although the distribution becomes diffuse as the lesion progresses [8]. It is known that FSGS has various etiologies and pathogenic mechanisms, being subdivided into primary, genetic, and secondary forms, all of which result in podocyte injury and depletion [9]. Clinically, it is characterized by proteinuria, microscopic hematuria, hypertension, and renal failure [10].

Renal function and proteinuria at presentation are considered prognostic factors in FSGS [11]. Patients with nephrotic proteinuria have a renal survival rate of 76% at 5 years and 57% at 10 years. The prognosis is more favorable in patients with non-nephrotic proteinuria, who have a renal survival rate of 92% at 10 years [12]. Because patients with FSGS and nephrotic syndrome classically have a worse prognosis, most studies have focused on the treatment and evolution of such patients, resulting in a lack of data for those without nephrotic syndrome.

The aim of this study was to evaluate patients with FSGS, comparing those with and without nephrotic syndrome at diagnosis in terms of the main etiologies and progression of renal disease.

## 2. Materials and Methods

This was a retrospective, observational, descriptive study in which we analyzed the epidemiological profiles of patients diagnosed with FSGS through renal biopsy, performed between 2009 and 2017 in the Nephrology Department of the Hospital das Clínicas of the University of São Paulo School of Medicine, in the city of São Paulo, Brazil. Clinical, laboratory, and histopathological (light microscopy and immunofluorescence) data were collected from medical records and from the internal electronic system for the visualization of examinations, thereafter being organized in a specific table.

### 2.1. Inclusion and Exclusion Criteria

We included all patients who were ≥15 years of age and were diagnosed with FSGS by renal biopsy during the study period. Patients with incomplete medical records were excluded, as were those for whom the renal biopsy data were missing.

### 2.2. Clinical and Laboratory Data

We assessed age and sex at the time of FSGS diagnosis, as well as the presence/absence of hypertension, presence/absence of hematuria, 24 h proteinuria or urinary protein/creatinine ratio, urea, creatinine, serum albumin, hemoglobin, and viral serology. Hematuria was defined as the presence of at least four red blood cells per field in two separate tests. Hypertension was defined as blood pressure higher than 140/90 mmHg in two measurements or the current or previous use of antihypertensive drugs [13]. For the purposes of this study, obesity was defined as a body mass index (BMI) ≥ 30 kg/m^2^.

Proteinuria, serum creatinine, and the use of immunosuppressive or non-immunosuppressive therapy were evaluated at 4 and 8 months after diagnosis, as was the glomerular filtration rate, estimated with the Chronic Kidney Disease Epidemiology Collaboration equation. The duration of immunosuppression was recorded, as was the occurrence of relapses and the treatments performed for them. Finally, we evaluated creatinine and proteinuria at the end of follow-up, as well as recording the length of the follow-up period for each patient.

Patients were divided into two groups—with and without nephrotic syndrome—and the groups were compared in relation to all of the variables evaluated. Nephrotic syndrome was defined by findings of edema, a 24 h proteinuria or urinary protein/creatinine ratio ≥ 3.5 g/day, and low serum albumin.

### 2.3. Renal Histology

In the analysis of renal biopsies, we accepted those including at least eight glomeruli, which were evaluated under light microscopy, with the Columbia classification being taken into account. In patients with non-nephrotic proteinuria at diagnosis and an unfavorable renal outcome, electron microscopy was employed in order to clarify the etiology and identify possible diagnostic errors. The following measures were taken to prepare the samples for electron microscopy.

The samples were deparaffinized by heating and then re-fixed in glutaraldehyde solution (2.5%) for 30 min. Subsequently, they were subjected to three 30 min washes in phosphate buffer. The tissue was then post-fixed for 2 h in 1.0% osmium tetroxide with 0.1 M cacodylate buffer. The tissue was again immersed in the buffer solution for three 15 min washes and a final rinse of 12 h. The dehydration of the tissue was followed by a series of washes in a graded (ascending) ethanol series at room temperature. The last step consisted of embedding the tissue in Spurr resin in an oven at 80 °C, with a curing period of 8 h in a silicone mold. After curing, the specimens were roughly trimmed to expose the portion containing the tissue of interest. The block was then positioned in an ultramicrotome and sectioned successively with a glass knife until the area of interest was identified. Ultrathin sections were then cut with a diamond knife to a thickness of 70–90 nm. The sections were expanded by exposure to a cotton swab soaked in chloroform and subsequently collected on a 100-mesh copper grid. Each grid was stained in two stages. With the specimen side down, the grids were immersed in uranyl acetate for 10 min and then rinsed in a series of four beakers of distilled water. After rinsing, the grids were stained with lead citrate for 15 min, rinsed again in distilled water, and stored in a grid box. Finally, electron micrographs were obtained with a 100 kV transmission electron microscope (Morgagni 268D; Philips/FEI, Hillsboro, OR, USA).

### 2.4. Assessment of Treatment Response—Remission Criteria

To assess the response to treatment and identify relapses, patients were evaluated at the 4th and 8th months after diagnosis, as well as at the end of the follow-up period. A complete response was defined as proteinuria ≤ 0.5 g/day and stable or improving serum creatinine, whereas a partial response was defined as proteinuria < 3.5 g/day, with a 50% decrease in relation to baseline and worsening in serum creatinine of ≤25% in relation to baseline. Patients who did not achieve complete or partial remission were categorized as nonresponders. Relapse was defined as having a complete or partial response at month 4 at diagnosis and a subsequent return, by month 8, to nephrotic-range proteinuria or a >50% increase in relation to the proteinuria at the time of the initial response.

### 2.5. Statistical Analysis

Numerical data are presented as the mean ± standard deviation or as the median and interquartile range (IQR). Categorical data are presented as absolute numbers and percentages. Comparisons of numerical data were performed between the two groups by using the unpaired *t*-test or Mann–Whitney test, as appropriate. Categorical data were compared by using Fisher’s exact test. Values of *p* < 0.05 were considered significant. Renal replacement therapy (RRT)-free survival in patients with and without nephrotic syndrome was analyzed by Kaplan–Meier curves. Multivariate Cox regression was employed to compare the patients who evolved to RRT with those who did not, and it was carried out only with the variables that presented a value of *p* < 0.20 in the univariate analysis. The statistical analysis was performed with the GraphPad Prism software, version 6.0 (GraphPad Software, Inc., San Diego, CA, USA), and SPSS 13.0.

## 3. Results

During the study period, 149 patients received a histological diagnosis of FSGS. Of these, nine patients were excluded because the data were incomplete. Therefore, the final sample comprised 140 patients. At diagnosis, 94 patients (67.1%) had nephrotic syndrome and 46 (32.9%) did not. As can be seen in Table 1, there was no significant difference between the patients in the nephrotic group and those in the non-nephrotic group in relation to the age at diagnosis, gender, and race distribution, as well as the estimated glomerular filtration rate and frequency of obesity. The prevalence of hypertension was significantly higher in the non-nephrotic group (82.0% vs. 39.3%, *p* < 0.0001), and hematuria was more common in the nephrotic group (68.0% vs. 47.8%, *p* = 0.006).

Table 1 also shows the histological types. The collapsing variant was more common in the nephrotic group (33.0% vs. 8.7%, *p* < 0.0001), as was the tip variant (11.7% vs. 2.1%, *p* = 0.005). In the non-nephrotic group, the most common histological type was the FSGS not otherwise specified (NOS) variant, which was observed in 82.6% of the patients, compared with only 51.0% of those in the nephrotic group (*p* < 0.0001). The perihilar variant was also more common in the non-nephrotic group (4.4% vs. 1.0%), although this difference was not significant (*p* = 0.17).

Of the 140 patients in our sample, 37 patients (26.4%) had a well-defined secondary form of FSGS. Infection with HIV alone was seen in 13 patients, three patients had diabetes mellitus, and two of these were also affected by HIV. Eight patients had systemic lupus erythematosus (SLE), of whom five were in the nephrotic group and were therefore diagnosed with lupus podocytopathy, and three were in the non-nephrotic group, considered as chronic lupus nephritis. Two patients had hepatitis C, and two had unclassified autoimmune disease. Other etiologies occurred in one patient each: infection with parvovirus B19; coinfection with hepatitis B and C; coinfection with HIV and hepatitis C; silicosis; anabolic steroid use; Alport syndrome; thrombotic microangiopathy; multiple myeloma; and preeclampsia. Among the 13 patients with HIV, nine were in the nephrotic group (four with the collapsing variant and five with the NOS variant). None of the four other HIV-affected patients (those in the non-nephrotic group) had the collapsing form.

### Treatment Data

Of the 94 patients in the nephrotic group, 67 (71.2%) received immunosuppressive therapy, 11 patients (11.7%) had no information about treatment, and 16 (17%) received no immunosuppressive therapy. Analyzing the initial immunosuppressive therapy employed, we found that some of these patients (*n* = 19) were treated with a regimen including prednisone alone; others (*n* = 28) were treated with calcineurin inhibitors, with or without prednisone; and the remaining patients (*n* = 20) were treated with various other regimens. Of the 19 patients treated with prednisone only, four (21%) achieved a complete response, three (15.7%) achieved a partial response, and 12 (63.1%) were categorized as nonresponders. Among the four patients who achieved a complete response after treatment with prednisone only, there were no occurrences of relapse in three, and one was lost to follow-up. Of the 28 patients treated with calcineurin inhibitors alone or calcineurin inhibitors with prednisone, eight (28.5%) achieved a complete response, five (17.8%) achieved a partial response, and 15 (53.5%) were categorized as nonresponders. Among the eight patients who achieved a complete response after treatment with calcineurin inhibitors, with or without prednisone, there were no occurrences of relapse in seven, and one was lost to follow-up. The other initial immunosuppressive therapy regimens were as follows: prednisone and mycophenolate mofetil in two patients; prednisone with cyclophosphamide in four; prednisone with azathioprine in one; and multiple immunosuppressive agents in 13. A complete response was achieved in one of the patients treated with these other immunosuppressive agents, six achieved a partial response, and 13 were categorized as nonresponders. In those who received no immunosuppressive therapy, a complete response was also achieved in one of the 16 patients, five achieved a partial response, and 10 were categorized as nonresponders.

As shown in Table 2, at eight months after diagnosis, the median proteinuria was higher in the nephrotic group than the non-nephrotic group (*p* = 0.03). However, there was no difference in serum creatinine between the groups (*p* = 0.56). The mean time on immunosuppression was 8.5 months, and the mean total follow-up period was 4 years and 10 months. By the end of follow-up, 11 of the patients in the nephrotic group had been lost to follow-up, leaving 83 patients, 26 (31.3%) of whom progressed to requiring RRT. In the non-nephrotic group, five patients were lost to follow-up, leaving 41 patients, of whom 11 (26.8%) progressed to RRT. In terms of the progression to RRT, the Kaplan–Meier curve analysis showed no statistical difference between the two groups, *p* = 0.35 (Supplementary Figure S1).

In the univariate analysis (Table 3), being a responder to immunosuppressive therapy was found to protect against progression to RRT in FSGS with nephrotic syndrome. In FSGS without nephrotic syndrome, having a final proteinuria level greater than 3.5 g/day was found to be an indicator of a worse prognosis and having proteinuria less than 1.5 g/day was protective. The histological types did not influence the progression to RRT, but there was a tendency for the collapsing form to have a worse prognosis and the tip variant to have a better prognosis, although this was not statistically significant. However, in the multivariate analysis, the assessments of the complete response, final proteinuria of less than 1.5 g/day or more than 3.5 g/day, and the collapsing and apical histological types, none of them showed statistical significance with the RRT outcome (Supplementary Table S1).

**Figure 1:**
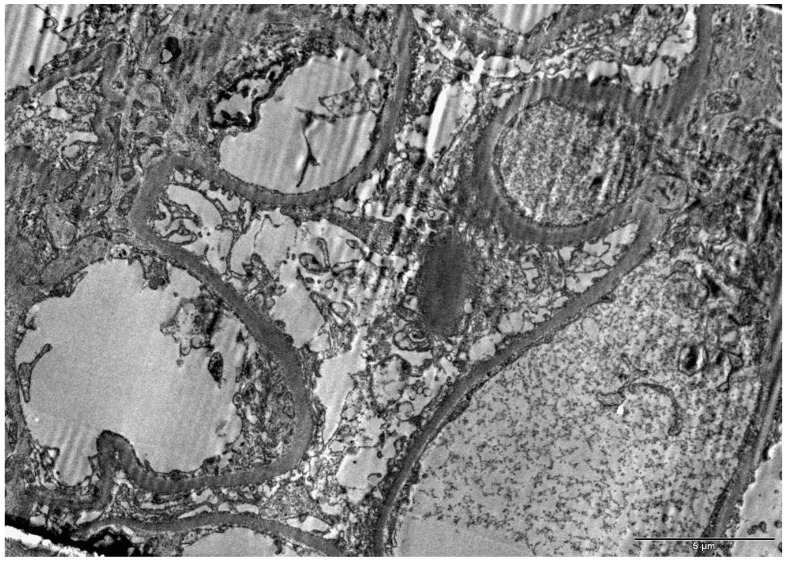
Electron micrograph of a renal tissue sample obtained from a patient with non-nephrotic proteinuria.

Among the 11 non-nephrotic group patients who progressed to RRT and had their samples evaluated by electron microscopy to rule out any diagnostic errors made in the evaluation by light microscopy and immunofluorescence, there were four cases in which the samples contained no glomeruli to be evaluated. In all seven of the remaining cases, the diagnosis of FSGS was confirmed by electron microscopy. One of the explanations for the lower renal survival rate in this subgroup of patients was a high degree of glomerular and tubulointerstitial chronicity, as seen on light and electron microscopy. Figure 1 shows an electron micrograph of a sample in which the glomerular characteristics resemble those of the other cases, including the absence of electron-dense deposits, a normal-looking basement membrane, and foot process effacement.

## 4. Discussion

The incidence of FSGS varies greatly depending on the place of residence and ancestry of the patient. Worldwide, the prevalence of FSGS has been increasing over the last two decades. This can be attributed to the increased availability of renal biopsies, significant population growth, changes in the diagnostic criteria, the greater prevalence of infections with viruses such as HIV and COVID, and an increase in the prevalence of obesity [3,4,5,14]. In this context of the increasing prevalence of a disease known for having a high rate of progression to stage 4 chronic kidney disease, in which the prognosis of FSGS depends on its clinical and histological presentation, as well as on its response to treatment, it becomes relevant to collect these data and evaluate the evolution of the affected patients.

In a study involving 338 patients with FSGS in the United States [15], 46% of whom presented nephrotic-range proteinuria at diagnosis, 27% of the patients in the sample as a whole evolved to requiring RRT within a mean time period of 9.5 years. The factors associated with this unfavorable evolution were high serum creatinine at diagnosis, high proteinuria, low serum albumin, a poor response to therapy, and a high degree of tubulointerstitial fibrosis on renal biopsy. In the present study, high proteinuria at diagnosis was not a factor related to a worse renal prognosis; we found the rate of progression to RRT to be similar between the patients with and without nephrotic syndrome in our sample. We believe that factors such as arterial hypertension and longer times between the onset of renal alterations and renal biopsy were more important for the lower renal survival in our non-nephrotic group. We assume that, because individuals with proteinuria in the non-nephrotic range tend to be asymptomatic, they also tend to wait longer for a histological diagnosis [12]. However, this purported delay was not evaluated in our study.

In a study of children diagnosed with FSGS, Yoshikawa et al. [16] compared those with and without nephrotic syndrome and concluded that both conditions had an unfavorable prognosis, with no difference between the two groups in relation to the renal outcome. However, in their histological analysis, the authors found that patients with the tip variant had a good renal prognosis, whereas approximately half of the patients with the perihilar variant evolved to the terminal stage of the disease [16]. In our study, we also found that none of the patients with the tip variant progressed to RRT.

What stands out in our study is the large number of patients with the collapsing variant of FSGS, which was seen in 35 cases, corresponding to 25% of the sample; as expected, the incidence of the collapsing variant was higher in the patients with nephrotic syndrome. Another study conducted in Brazil and excluding HIV-affected patients demonstrated a high frequency of the collapsing variant of FSG [17]. In that study, 36.6% of the patients had the collapsing variant, and the authors compared this finding with the 11% reported in a study conducted in the United States (in the state of North Carolina). There is still no clear explanation for the higher frequency of this histological variant in Brazil. It might be due to the improvement in the quality of histological diagnoses and changes in the epidemiological behavior of its associated conditions, such as infections, autoimmunity, neoplasia, and genetic factors such as the APOL1 genotype. Therefore, the population of Brazil must present genetic and epidemiological factors that favor the development of collapsing glomerulopathy [18].

In the analysis of the glomerular ultrastructure in FSGS, the expected findings on electron microscopy are the absence of electron-dense deposits and the presence of foot process effacement [19]. Various diseases can present as FSGS under light microscopy, especially without the manifestation of nephrotic syndrome, such as Fabry disease and Alport syndrome, with electron microscopy being necessary to confirm these two diagnoses. Therefore, the objective of performing electron microscopy in the 11 patients without nephrotic syndrome who progressed to RRT was to determine whether Fabry disease or Alport syndrome had been misdiagnosed as FSGS, which was not found to be the case in any of the patients in our sample. The progression to dialysis in these 11 patients could still be attributed to genetic forms of FSGS, which have been associated with worse renal survival even in the absence of nephrotic syndrome [20]. However, no genetic testing was performed in the present study.

In 26.4% of our sample, we found an association with well-defined secondary causes. In both groups, the two main associated etiologies were SLE and infection with HIV. We could also conjecture an association with hypertension, which was more prevalent in the non-nephrotic group. In a study of 46 patients with FSGS in the United States, the frequency of secondary causes was 67%, higher than in our study, although adaptive FSGS was more common among the patients evaluated in that study [21]. In our study sample, the incidence of obesity was 16.4% and did not differ significantly between the nephrotic and non-nephrotic groups.

In the present study, unfavorable renal outcomes were more common among the nephrotic group patients who did not respond to immunosuppressive therapy than among those who achieved a partial or complete response. This was also the case for the non-nephrotic group patients who had proteinuria above 3.5 g/day at the end of follow-up. However, in an ongoing cohort study of patients with evidence of proteinuria at the time of their first kidney biopsy, the renal outcomes were better among the patients who achieved a partial response but had proteinuria < 1.5 g/day [22]. Therefore, attempts should always be made to reduce proteinuria during the evolution of the disease, because FSGS with proteinuria of 2.5 g/day will certainly evolve differently compared to FSGS with proteinuria < 1.5 g/day. Although this is relevant information for clinical practice, other studies have found that, in patients with FSGS who achieved a partial response, there were no differences in terms of renal survival among the subgroups with proteinuria < 1.0 g/day, 1.0–2.0 g/day, and 2.0–3.5 g/day [11].

The patients in our study who used multiple immunosuppressive regimens were associated with multiple relapses, and, in seven of them, rituximab was used. A study conducted on 183 adult patients with FSGS and minimal change disease, whose response profile to corticosteroids was 68% steroid-dependent, showed that the use of rituximab, especially as maintenance therapy, showed a longer relapse-free time compared to those without maintenance treatment [23].

## 5. Conclusions

In this study, which included a large number of patients with a biopsy-confirmed diagnosis of FSGS, the proportion of patients in whom there was an unfavorable renal outcome did not differ between those with nephrotic syndrome in the initial clinical presentation and those without. Factors related to better renal survival were having had a complete response to treatment in the case of those with nephrotic syndrome and having achieved proteinuria values of less than 1.5 g/day in the case of those without nephrotic syndrome. Our findings encourage further studies aimed at improving renal survival in patients with this disease.

## Figures and Tables

**Table 1:** Clinical and histological presentation of focal segmental glomerulosclerosis at diagnosis *.

Variable	Nephrotic Syndrome		*p*
	With	Without	
	*n* = 94	*n* = 46	
Age (years)	37.00 (24.75–54.25)	31.00 (25.00–44.75)	0.33
Male, *n* (%)	53 (56.4)	21 (45.6)	0.20
Black, *n* (%)	28 (29.7)	11 (23.9)	0.52
Hemoglobin (g/dL)	12.10 (10.70–13.70)	13 (11.20–14.23)	0.06
Hypertension, *n* (%)	37 (39.3)	38 (82.0)	<0.0001
BMI > 30 kg/m^2^, *n* (%)	15 (15.9)	8 (17.3)	1
Serum creatinine (mg/dL)	1.45 (0.88–2.13)	1.34 (0.92–2.15)	0.93
eGFR (mL/min/1.73 m^2^)	56.00 (30.00–103.50)	46.00 (30.50–73.50)	0.15
Serum albumin (g/dL)	1.80 (1.40–2.40)	3.60 (3.30–4.02)	<0.0001
Proteinuria (g/day)	6.53 (4.60–9.77)	2.63 (1.62–3.85)	<0.0001
Hematuria, *n* (%)	64 (68.0)	22 (47.8)	0.006
Histological type, *n* (%)			
Not otherwise specified	48 (51.0)	38 (82.6)	<0.0001
Collapsing	31 (33.0)	4 (8.7)	<0.0001
Perihilar	1 (1.0)	2 (4.4)	0.17
Cellular	3 (3.2)	1 (2.1)	0.65
Tip	11 (11.7)	1 (2.1)	0.005

BMI, body mass index; eGFR, glomerular filtration rate estimated with the Chronic Kidney Disease Epidemiology Collaboration equation. * Except where otherwise indicated, data are presented as the median (interquartile range).

**Table 2:** Clinical evolution of patients with focal segmental glomerulosclerosis, with and without nephrotic syndrome *.

Variable	Nephrotic Syndrome		*p*
	With	Without	
	*n* = 94	*n* = 46	
Proteinuria 8 months after diagnosis (g/day)	3 (0.72–5.10)	1.24 (0.50–3.10)	0.03
Creatinine 8 months after diagnosis (mg/dL)	1.20 (0.83–2.19)	1.36 (0.93–2.21)	0.56
RRT at the end of follow-up, *n* (%)	26 (31.3) *	11 (26.8) ^†^	0.44
Follow-up time (years)	4.00 (2.00–7.00)	5.00 (2.25–7.75)	0.19

RRT, renal replacement therapy. * Data available for only 83 patients. ^†^ Data available for only 41 patients.

**Table 3:** Comparison between patients who progressed to renal replacement therapy by the end of follow-up and those who did not.

Variable	RRT Required		*p*
	Yes	No	
	*n* = 37	*n* = 87	
With nephrotic syndrome, *n*	26	57	
Complete response, *n* (%)	0 (0)	14 (24.5)	0.0038
Partial response, *n* (%)	8 (30.7)	11 (19.3)	0.2705
No response, *n* (%)	18 (69.2)	32 (56.1)	0.3356
Without nephrotic syndrome, *n*	11	30	
Final proteinuria < 1.5 g/day, *n* (%)	2 (18.1)	18 (60)	0.0325
Final proteinuria of 1.5–3.5 g/day, *n* (%)	2 (18.1)	8 (26.6)	0.7004
Final proteinuria > 3.5 g/day, *n* (%)	7 (63.6)	4 (13.1)	0.0031
Histological type, *n* (%)			
Not otherwise specified	21 (56.7)	56 (64.3)	0.4275
Collapsing	14 (37.8)	18 (20.6)	0.0713
Perihilar	1 (2.7)	2 (2.3)	1
Tip	0 (0)	9 (10.3)	0.0565
Cellular	1 (2.7)	2 (2.3)	1

RRT, renal replacement therapy.

## Data Availability

All data generated or analyzed during this study are included in this article. Further inquiries can be directed to the corresponding author.

## Supplementary Materials

The following supporting information can be downloaded at: https://www.mdpi.com/article/10.3390/diagnostics15020120/s1, Figure S1: Renal survival comparing nephritic versus non-nephrotic group; Table S1: Multivariate analysis of data from patients with and without nephrotic syndrome, comparing those who evolved to needing renal replacement therapy with those who did not.
