# Supplementary material for: Nephrotic and Non-Nephrotic Focal Segmental Glomerulosclerosis: Clinical Characteristics, Etiology, and Columbia Classification
Source: Diagnostics (Basel). 2025 Jan 7;15(2):120. doi: 10.3390/diagnostics15020120 (PMC11763650; doi:10.3390/diagnostics15020120)
Supplement: Supplementary file 1 [file diagnostics-15-00120-s001.zip › diagnostics-3381763-supplementary.pdf]

Supplemental material

Figure S1. Renal survival comparing nephrotic versus non-nephrotic group

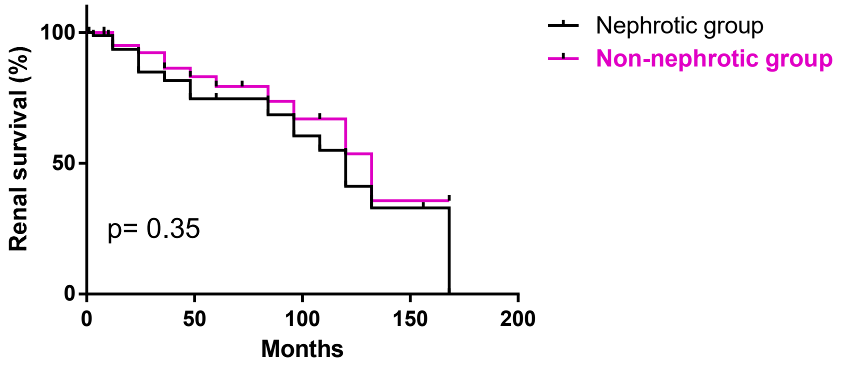

Table S1. Multivariate analysis of data from patients with and without nephrotic syndrome, comparing those who evolved to needing renal replacement therapy with those who did not

	B	Wald	p
Complete response	**11.72**	**0.014**	**0.907**
Final proteinuria < 1.5 g/day	**1.10**	**2.409**	**0.121**
Final proteinuria > 3.5 g/day	**-0.17**	**0.122**	**0.953**
Histological type, Collapsing	**11.65**	**0.003**	**0.953**
Histological type, Tip	**10.30**	**0.003**	**0.954**
